# Training Facilities in Little Hospitals

**Published:** 1919-10-11

**Authors:** 


### THE MATRONS' AND SISTERS' DEPARTMENT.

### TRAINING FACILITIES IN LITTLE HOSPITALS.

The notion of co-operation is gaining ground in the training-schools. Not so very long ago matrons of first-class hospitals looked askance at any probationers who could boast of "previous experience." It was a fatal drawback to candidates to have put in a year or two at a children's, special, or fever institution. They had so much to unlearn, they were told, and would do better to apply elsewhere. All this has changed in a wonderful manner. And this not altogether on account of the new shortage of candidates. Times have altered, and even the smallest hospitals enjoy the superintendence of matrons from good schools. Their routine is conducted on orthodox lines, and the pupils who leave them to commence serious training have by no means anything to unlearn. On the contrary, they are often so well prepared for their duties that ward sisters have been known to fight for them. The leading hospitals both in London and the North have now a wide-reaching system of affiliation through which they covenant to receive probationers from special hospitals known to them as affording good preliminary training and to shorten the term of service from four to three years for such as are suitable. In this way the Metropolitan Asylums Board is enabled to pass on its probationers to complete their training, St. Bartholomew's, King's, the Royal Free, Sheffield Royal Infirmary, Bristol General Hospital, and the Devon and Exeter Hospital all having agreed to allow two years of fever training in an M.A.B. institution to count as one.

Considering the success which has attended these and similar efforts at co-operation, it may be inquired why the system has not been further extended to link quite little hospitals with such moderate-sized general training-schools as grant a certificate after three years' work. The number of hospitals containing under twenty beds is increasing every day. Many competent authorities consider they may help appreciably to solve the problem of hospital congestion. They are being founded all over the country now as war memorials, and are welcomed by local medical men on two main grounds. The first is the grave difficulty of securing beds on the medical side in the county hospitals, where surgical cases are invariably preferred. The second is the urgent need which exists in all the smaller towns for paying beds suitable for persons unable to pay the fees of nursing-homes run for profit.

All these little hospitals require probationers. None of them is qualified to give any training which could count as such in the making of a nurse. The question to be answered in their connection is whether they can be made of any use in the general nurse-training system of the country. We believe that, wisely handled, the women who are employed as aides to the matron and staff nurses in these little institutions may become a considerable source of strength to the larger institutions in the same county, whether under municipal or voluntary control. All that is required is a system of standardisation and a disposition to open the eyes to the modern aspirations of the candidates.

We have seen women, awkward, very nervous, without a grain of self-confidence, pass within a few months out of a tiny hospital eager to become trained, self-reliant, and fully prepared for the duties they will be called on to fulfil on first entering a hospital. No better preparation could be devised for candidates than to serve in a tiny institution under a capable matron prior to commencing their training. The main need is for a definite course to be laid down which the candidate, during this period, can be put through. To have such a course indicated would be the greatest possible aid to the matrons of little institutions, because it would stimulate workers and help them to feel from the beginning that they were not in a back-water, but were part of the great nursing world. Some pupils would enter for six months, some for a year. Both practical and theoretical nursing would find a place in the easy curriculum. But the main gain to the pupils must always lie in that individual training of their faculties, best carried out by a trained woman herself working side by side with her helpers. In our judgment such preparation might in some respects exceed in value that given in the preliminary schools, though we would not be thought to depreciate these well-equipped centres of instruction. In the first place, the training would cost nothing either to the hospital or to the pupil. In the next place, the pupil would be of actual use to the sick from the beginning. In the third place, she would receive her preliminary lessons under conditions approximating to those she will find in the wards. Her patients would be no artfully designed dolls; her domestic duties would be essential to the welfare of the household; she would feel herself to be a responsible worker from the beginning.

